# Mucoadhesive Hydrogel Films of Econazole Nitrate: Formulation and Optimization Using Factorial Design

**DOI:** 10.1155/2014/305863

**Published:** 2014-06-10

**Authors:** Balaram Gajra, Saurabh S. Pandya, Sanjay Singh, Haribhai A. Rabari

**Affiliations:** ^1^Ramanbhai Patel College of Pharmacy, Charotar University of Science and Technology, CHARUSAT Campus, Changa, Petlad Taluka, Anand District, Gujarat State 388421, India; ^2^Shree Krishna Institute of Pharmacy, Krishna Campus, Shankhalpur, Bechraji Taluka, Mehsana District, Gujarat State 384210, India; ^3^Department of Pharmaceutics, Indian Institute of Technology, Banaras Hindu University, Varanasi, Uttar Pradesh State 221005, India; ^4^L.M. College of Pharmacy, Navrangpura, Ahmedabad, Gujarat State 380009, India

## Abstract

The mucoadhesive hydrogel film was prepared and optimized for the purpose of local drug delivery to oral cavity for the treatment of oral Candidiasis. The mucoadhesive hydrogel film was prepared with the poly(vinyl alcohol) by freeze/thaw crosslinking technique. 3^2^ full factorial design was employed to optimize the formulation. Number of freeze/thaw cycles (4, 6, and 8 cycles) and the concentration of the poly(vinyl alcohol) (10, 15, and 20%) were used as the independent variables whereas time required for 50% drug release, cumulative percent of drug release at 8th hour, and “*k*” of zero order equation were used as the dependent variables. The films were evaluated for mucoadhesive strength, in vitro residence time, swelling study, in vitro drug release, and effectiveness against *Candida albicans*. The concentration of poly(vinyl alcohol) and the number of freeze/thaw cycles both decrease the drug release rate. Mucoadhesive hydrogel film with 15% poly(vinyl alcohol) and 7 freeze/thaw cycles was optimized. The optimized batch exhibited the sustained release of drug and the antifungal studies revealed that the drug released from the film could inhibit the growth of *Candida albicans* for 12 hours.

## 1. Introduction

Oral candidiasis is an infectious condition caused by the fungus of the genus* Candida*, the most common of which is* Candida albicans*. Oral candidiasis significantly occurs to the immunocompromised individuals, namely, patients with AIDS immunosuppression, cancer chemotherapy/radiation therapy, corticosteroid therapy, and chronic antibiotic therapy and individuals with xerostomia and diabetes mellitus [1, 2].

Chronic systemic administration of antifungal agents is required to treat the oral candidiasis, which may produce potential adverse effects. Therefore local administration of the antifungal agent is considered as the first line treatment for the oral candidiasis if the drug concentration remained above the minimum inhibitory concentration throughout the treatment [3]. It is difficult to achieve by the conventional dosage forms like oral gels, pastes, solution, suspensions, ointments, mouthwashes, lozenges, mouth paints, and so forth, because less retention time of the dosage form leads to the fluctuation in the salivary drug concentration [1, 2]. Therefore an ideal dosage form for the treatment of the oral candidiasis is one which sustains the release of drug and retains in the oral cavity and produce antifungal effect for prolonged period of time [4]. This is possible if the drug delivery system possesses sustained release as well as mucoadhesive properties [5].

Imidazole antifungals such as miconazole, clotrimazole, itraconazole, and econazole are the major drugs used for the treatment of oral candidiasis. Delivery of the antifungals like miconazole to the oral mucosa has been investigated by various workers exploiting novel drug delivery systems like nanostructured lipid carriers [6].

Econazole nitrate (ECN) is an imidazole antifungal agent which is effective against* Candida albicans*. The ECN interferes with the ergosterol synthesis and thereby alters the normal functions of the cell membrane and causes death of the fungus. ECN is investigated for the topical administration to the skin [7–9], vaginal [10–12], and buccal applications [13, 14] for the treatment of fungal infections.

Poly(vinyl alcohol) (PVA) hydrogels are extensively studied for controlled drug release for various drugs [15–19]. PVA hydrogels must be crosslinked to impart the controlled and sustained release of drugs [20]. The cross linking density of the PVA hydrogel decreases the rate of drug release. Chemical crosslinking of PVA hydrogel may lead to the toxic impurities. When PVA is exposed to cycles of freezing and thawing, it gets crosslinked [17, 21]. Freeze/thaw method has many advantages including nontoxicity in comparison to the chemical method [22]. The PVA hydrogels prepared by freeze/thaw technique possess mucoadhesive property [15].

Statistical optimization techniques are frequently employed for the development of pharmaceutical formulations [23–25]. 3^2^ full factorial design is the simple experimental design with two variables studied at three levels [25].

The objective of the present work was to develop mucoadhesive hydrogel film (MHF) of ECN using PVA hydrogel exploiting freeze/thaw technique as crosslinking method. 3^2^ full factorial design was used to optimize the effect of concentration of PVA and number of freeze/thaw cycles on drug release. The optimized batch was prepared and studied for physical parameters as well as antifungal efficacy.

## 2. Materials and Methods

### 2.1. Materials

Econazole nitrate (gift sample from Gufic Biosciences Ltd., Navsari, Gujarat, India); Poly(vinyl alcohol) (Fisher Scientific-Qualigens fine chemicals, Navi Mumbai, India); Polyethylene Glycol 400 (Merck Ltd., Mumbai, India); Sabouraud Dextrose Agar (SDA) with chloramphenicol (Hi-Media, Mumbai, India) Ultrapure water (Millipore, USA) were used throughout and all the other chemicals used were of analytical grade.

### 2.2. Methods

#### 2.2.1. Preformulation and Drug-Polymer Interaction Studies

Drug-polymer and polymer-polymer interaction studies were done by Differential Scanning Calorimetry and Fourier Transform Infrared spectroscopy.

*Differential Scanning Calorimetry (DSC).* A differential scanning calorimeter (model Pyris-1 DSC, Perkin Elmer, USA) was used to determine drug-polymer interaction studies. Approximately 65 mg of the pure drug, polymer alone, drug-polymer physical mixture (1 : 1), and the formulated MHF was weighed and then vacuum dried for 24 hours to remove all the water. The dried sample (16.5 mg) was heated in the DSC equipment from 45 to 400°C at a scanning rate of 10°C/min in the nitrogen atmosphere. The heat required to melt the sample was calculated by integrating the peak due to melting. Thermograms obtained were compared to interpret any drug-polymer interaction.

*Fourier Transform Infrared (FTIR) Spectroscopy.* An FTIR spectrophotometer (Model Spectrum GX with IR Quant software, Perkin Elmer, USA) was used for the study. Pure drug, polymer alone, drug-polymer physical mixture (1 : 1), and formulated MHF were subjected to FTIR spectroscopy in the range of 4000 to 400 cm^−1^. The peaks of pure drug were compared with the physical mixture and the MHF formulations to interpret any drug-polymer interaction.

#### 2.2.2. Method of Preparation of Mucoadhesive Hydrogel Films (MHFs)

PVA dissolved in double distilled water at 90°C with different concentrations for 6 hours by continuous stirring on magnetic stirrer with hot plate. Accurately weighed ECN dissolved in the mixture of ethanol and polyethylene glycol 400 (PEG400) (3 : 5) and the resulting solution of ECN was added to the PVA solution by continuous stirring for 30 minutes at room temperature. The drug containing aqueous solution of PVA was poured in to the glass mould of 10 × 10 cm. The gel containing drug was cross linked by freezing the mixtures in mould at −12°C for 16 hr followed by thawing at room temperature for 8 hrs, that is, by freeze/thaw technique. The freeze/thaw cycle was repeated for four, six, and eight times. The resulting crosslinked and drug entrapped hydrogel was dried at room temperature to get the solid films. The hydrogel films were cut into the pieces of 1.5 × 1.5 cm. The amount of ECN was added in such a manner that the area of 2.25 cm^2^; that is, one film contains 80 mg drug. The resultant hydrogel films were packed individually in aluminium foil and stored in a desiccator at room temperature. The details of the formulation are given in Tables 1, 2, and 3.

#### 2.2.3. Morphological Evaluation of MHFs

The MHFs were observed for their colour by the naked eyes in the day light.

Scanning electron microscopy (SEM) was used to study the surface morphology of the prepared MHFs. Samples (MHFs) were attached to metal stubs which had been previously covered with coated tape and then sputtered with a layer of gold. They were examined with a scanning electron microscope (Model ESEM EDAX XL-30, Philips, Netherlands). The images were taken at 500x and 1000x magnification.

#### 2.2.4. Drug Content Uniformity Studies

Five randomly selected MHFs containing ECN were dissolved in 100 mL methanol by stirring continuously for 2 hours on magnetic stirrer and filtered by filter paper. The amount of drug in the solution was then measured spectrophotometrically at 225 nm. The average of five MHFs was reported.

#### 2.2.5. Physical Properties of MHFs

*Microenvironment pH.* The microenvironment pH of the prepared MHFs was determined to evaluate the possible irritation effects on the mucosa. The films were left to swell in 5 mL of pH 6.8 phosphate buffer in small beakers and the pH was measured after one hour by placing the electrode in contact with the microenvironment of the swollen films. The average pH of five determinations was reported [1, 26].

*Thickness.* The thickness of MHFs was determined by standard screw gauge. The average thickness of five MHFs was reported [1, 26].

*Weight.* MHFs were weighed by standard electronic balance. The average weight of five MHFs was reported [1, 26].

*Folding Endurance.* Folding endurance was determined on five MHFs in each batch by repeatedly folding the film at the same place till it broke or folded up to 300 times [1, 26].

#### 2.2.6. Moisture Absorption

Moisture absorption (MA) study was performed by a modified method of Singh et al. and Kanig and Goodman [1, 27]. Accurately weighed preconditioned films (*W*_0_) were placed in a constant humidity chamber (containing a saturated solution of ammonium chloride which gives a relative humidity of 79.5%) set at room temperature. Films were weighed (*W*_*t*_) at an interval of 1, 3, and 7 days. The average MA of three MHFs was reported.

Percent MA was calculated using the following equation:

(1) $$\begin{array}{c} \%\text{MA}=\left[\frac{W_{t}-W_{0}}{W_{0}}\right]\times 100. \end{array}$$

#### 2.2.7. In Vitro Mucoadhesive Studies

The method developed by Singh et al. and Kim et al. [1, 2, 28, 29] was slightly modified to study the bioadhesive character of the prepared MHFs. The apparatus used for study comprised two-arm balance, one side of which contains two glass plates and other side contains a container.

One glass plate was attached with the base of the balance and other with the arm of the balance by a thick strong thread. The membrane used for mucoadhesive testing was fresh goat buccal mucosa. The buccal mucosa of goat was collected from the slaughter house. Fresh goat buccal mucosa was glued to the upper side of the lower plate and MHF was glued to the lower side of the upper plate by using Cyanoacrylate adhesive (Superwiz). Then 50 gm preload force (or contact pressure) was applied on upper plate for 5 minutes (preload time). After removal of the preload force, the water kept in a bottle at some height was siphoned in the container at the rate of 10 mL per minute till the plates were detached from each other. The rate of dropping of water was controlled with on-off switch same as in infusion bottle. The weight of water required for detachment of glass plates was considered as the mucoadhesive strength in mg/cm^2^ of the MHF under study. The average mucoadhesive strength of three MHFs was reported.

#### 2.2.8. Water Uptake or Swelling Study

*Procedure*. The water uptake or swelling index of the MHF was determined by equilibrium weight gain method similar to that reported by Efentakis and Vlachou [30] and Roy and Rohera [31]. The study was carried out using USP/NF dissolution apparatus I. The MHFs were accurately weighed, placed in dissolution baskets, and immersed in pH 6.8 phosphate buffer maintained at 37 ± 1°C in the dissolution vessels. At regular intervals (0.5, 1, 2, 3, 4, 5, 6, 7, 8, 9, 10, 11, and 12 hours), the preweighed basket with MHF was withdrawn from the dissolution vessel, lightly blotted with a tissue paper to remove excess test liquid, and reweighed. The percent swelling index or water uptake was estimated at each time point using the following equation:

(2) $$\begin{array}{c} \%S=\left[\frac{X_{t}-X_{o}}{X_{p}}\right]\times 100, \end{array}$$

where *X*_*t*_ is the weight of the swollen MHF after time *t* and *X*_*o*_ is the original MHF weight at zero time and *X*_*p*_ is the amount of polymer in the MHF.

The water uptake or swelling index data was mean of three determinations.

*Model Fitting*. The water uptake data was subjected to the Vergnaud model to determine the rate of water uptake [32]. The generalized form of the Vergnaud model is as follows [33]:

(3) $$\begin{array}{c} M_{t}={kt}^{n}, \end{array}$$

where *M*_*t*_ represents the amount of liquid transferred at time *t* and *k* is swelling constant which depends on the amount of liquid transferred after infinite time, the porosity of matrix, and diffusivity. The exponent, *n*, indicates the mechanism of water uptake.

The values of *n* and *k* were determined by converting the equation to the logarithm form as follows:

(4) $$\begin{array}{c} \text{log}\left(M_{t}\right)=\log\left(k\right)+n\log\left(t\right) \end{array}$$

and plotting the curve log⁡⁡(*M*_*t*_) versus log⁡⁡(*t*). The values of slope and intercept so obtained by plots are *n* and log⁡⁡(*k*), respectively.

#### 2.2.9. Determination of the In Vitro Residence Time

The in vitro residence time was determined using a locally modified USP tablet disintegration testing apparatus, based on the apparatus applied by Nakamura et al. [34]. The medium was composed of 700 mL phosphate buffer (pH 6.8) in 1 L glass beaker and maintained at 37 ± 1°C. A segment of goat buccal mucosa, 2.5 × 2.5 cm, was cut and glued by cyanoacrylate adhesive (Superwiz) on the inside curved surface of 1 L beaker above the level of 700 mL phosphate buffer (pH 6.8). A glass cylinder (100 mL) was vertically fixed to the apparatus. The MHF was hydrated from one surface using phosphate buffer (pH 6.8) and then the hydrated surface was brought into contact with the mucosal membrane. The glass cylinder was vertically fixed to the apparatus and allowed to move up and down so that the film was completely immersed in the buffer solution at the lowest point and was out at the highest point. The time necessary for complete detachment of the MHF from the mucosal surface was recorded (mean of triplicate determinations).

#### 2.2.10. In Vitro Drug Release Study

The drug release studies were carried out using a US Pharmacopoeia XXX (USPXXX) type II dissolution apparatus modified to the USPXXX dissolution apparatus type V, that is, paddle over disc apparatus [35]. The dissolution was done at 37 ± 1°C and 50 rpm with pH 6.8 phosphate buffer containing 1% SLS as a medium (900 mL) [3]. The MHF was fixed with a cyanoacrylate adhesive (Superwiz) to a thick glass disk placed at the bottom of the vessel of the dissolution testing apparatus containing deaerated, prewarmed (37°C ± 0.1°C) media. The 3 mL sample was withdrawn in the time interval of 1, 2, 3, 4, 6, 8, 10, and 12 hours. The media was replaced with same amount of fresh media. The collected samples were filtered and the amount of drug was measured spectrophotometrically using UV-Visible spectrophotometer at 305 nm. The mean of three triplicate determinations was reported.

The release profiles were fitted in zero order, Korsmeyer-Peppas equation, Higuchi equation, and first order kinetics to determine the mechanism of drug release.

#### 2.2.11. Antifungal Efficacy (of Optimized MHFs)

*Preparation of the Agar Plates*. The agar plates used in this study were prepared by dissolving Sabouraud Dextrose Agar (SDA) 65 g in 1 L of distilled water and sterilized by autoclaving (at 15 lb pressure and 121°C) for 15 min. The agar solution was poured into sterile petri dishes. The agar plates were then allowed to cool and solidify at room temperature; then they were inoculated (cultured) with* C. albicans* by using a sterile swab [36].

*Standard Calibration Curve of ECN.* Standard dilutions of 5 to 80 *μ*g/mL of ECN were prepared in pH 6.8 phosphate buffer containing 1% SLS. A 0.1 mL of each dilution was carefully pipetted into 7 mm diameter well of the agar plate. These plates were allowed to prediffuse for 2 hr at room temperature and then incubated for 24 hr. The diameter (mm) of the growth inhibition zone surrounding each agar well inoculated with* C. Albicans* was measured and standard calibration curve of concentration of drug versus diameter of zone of inhibition (mm) was plotted. The mean of three determinations of each sample was determined.

*Agar Diffusion Assay of MHFs. *Antifungal efficacy of the optimized MHFs was determined by subjecting the aliquots of in vitro drug release studies to disc agar diffusion assay [36, 37]. Aliquots of in vitro drug release samples were collected at 1, 2, 3, 4, 6, 8, 10, and 12 hour. A 0.1 mL of each sample was carefully pipetted into uniformly spaced 7 mm diameter wells of the agar plates. These plates were allowed to prediffuse for 2 hours at room temperature and then incubated for 24 hour. The diameter (mm) of the growth inhibition zone surrounding each agar well inoculated with* C. Albicans* was measured, and the concentrations of ECN were determined from the standard calibration curve constructed under identical conditions, ranging from 5 to 80 *μ*g/mL. The mean of three determinations of each sample was determined.

*Statistical Analysis*. For comparison among the batches, one way ANOVA was used with *P* value of less than 0.05 to consider as evidence of a significant difference.

3^2^ full factorial design and the regression analysis were used to optimize the concentration of PVA and number of freeze/thaw cycles.

By using the independent variables and their degrees as well as the values of dependent variables obtained by the experiments, the following type of regression equation was generated for each dependent variable. A statistical model incorporating interactive and polynomial terms was utilized to evaluate the response:

(5) Y=β0+β1X1+β2X2+β12X1X2 +β11X12+β22X22,

where *Y* is the dependent variable, *β*_0_ is the arithmetic mean response of the nine runs, and *β*_1_ and *β*_2_ are the estimated coefficient for the factors *X*_1_ and *X*_2_. The main effects (*X*_1_ and *X*_2_) represent the average result of changing one factor at a time from its low to high value. The interaction terms (*X*_1_ *X*_2_) show how the response changes when two factors are simultaneously changed. The polynomial terms (*X*_1_^2^ and *X*_2_^2^) are included to investigate nonlinearity.

## 3. Results and Discussion

### 3.1. Preformulation and Drug-Polymer Interaction Studies

#### 3.1.1. Differential Scanning Calorimetry (DSC)

**Figure 1:**
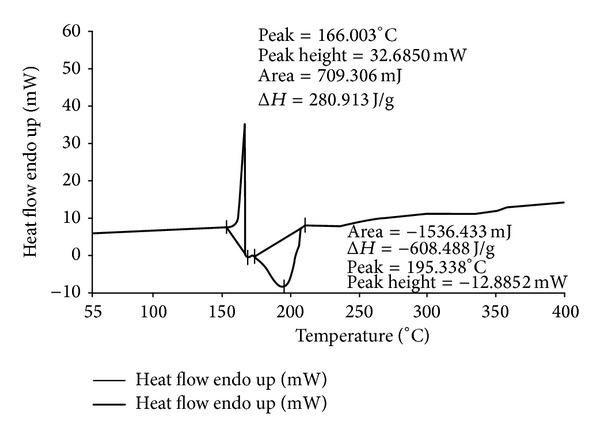
DSC thermogram of econazole nitrate pure.

**Figure 2:**
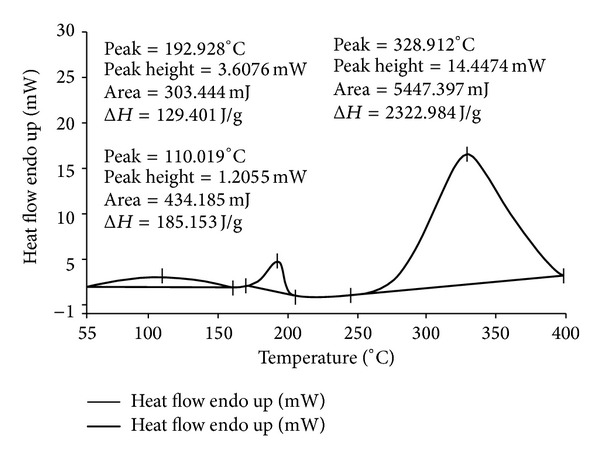
DSC thermogram of PVA pure.

**Figure 3:**
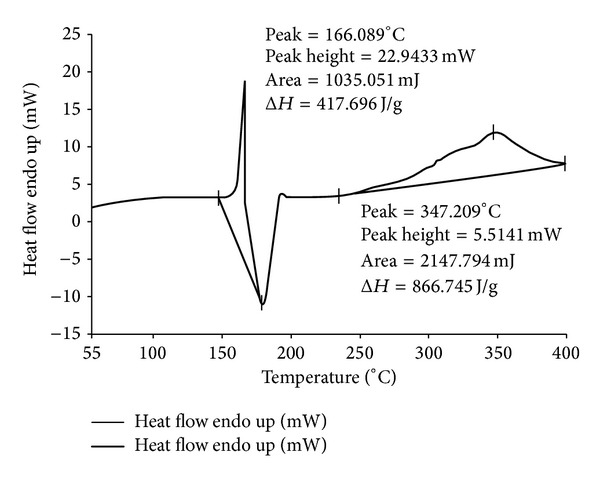
DSC thermogram of econazole nitrate : PVA (1 : 1).

**Figure 4:**
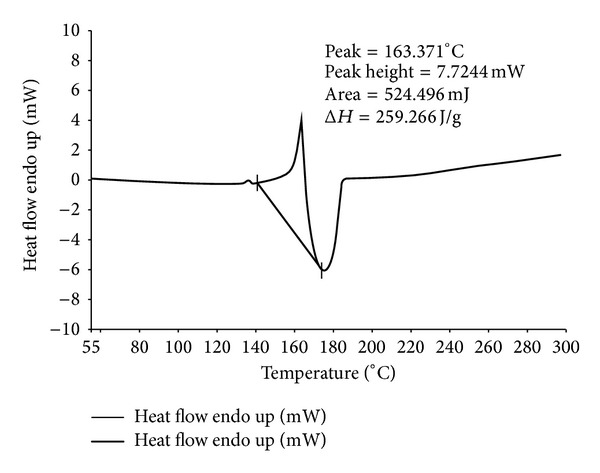
DSC thermogram of MHF.

The thermograms of ECN pure drug, PVA pure, 1 : 1 physical mixture of ECN with PVA, and MHF are given in Figures 1, 2, 3, and 4. ECN has two peaks in the thermogram, one positive (endothermic) peak at 166.003°C with the peak area of 709.306 mJ and another negative peak at 195.338°C with the peak area of −1536.433 mJ (Figure 1). The reason behind the endothermic peak at 166.003°C is the melting of ECN (melting range of ECN is 161 to 166°C). Both the peaks were found intact in the thermograms of 1 : 1 physical mixture of ECN with PVA as well as in the thermogram of MHF. This confirms the absence of chemical interaction of drug with the polymers.

#### 3.1.2. Fourier Transform Infrared Spectroscopy (FTIR)

**Figure 5:**
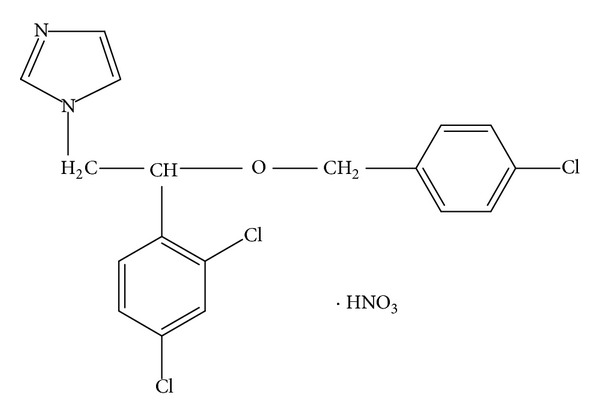
Chemical structure of econazole nitrate.

**Figure 6:**
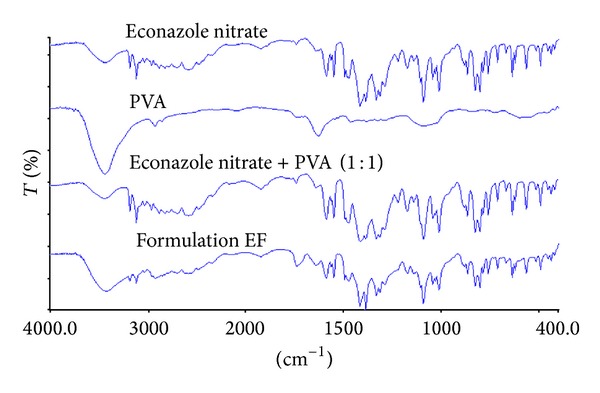
FTIR spectra of econazole nitrate pure, PVA pure, econazole nitrate : PVA physical mixture (1 : 1), and MHF.

The FTIR spectra of ECN pure drug, PVA pure, 1 : 1 physical mixture of ECN with PVA, and MHF are given in the Figure 6. The FTIR spectra showed the prominent peaks of the various bonds between the groups present in the ECN chemical structure (Figure 5). The prominent peaks for various groups are N–H stretching at 3426 cm^−1^, C–Cl stretching at 638 cm^−1^, C–H stretching for aromatic at 3109 cm^−1^, C=C stretching for aromatic at 1585 cm^−1^, C–N stretching at 1330 cm^−1^, C–O stretching for ether at 1090 cm^−1^, and –NO_2_ at 1547 cm^−1^. It was observed that all the above stated prominent peaks of the ECN pure were present in the FTIR spectra of 1 : 1 physical mixtures of ECN with PVA and MHF. This confirms that there was no chemical interaction between the drug and the polymers or confirms that the drug is present in pure and unchanged form in the MHF.

### 3.2. Morphological Evaluation of Hydrogels

**Figure 7:**
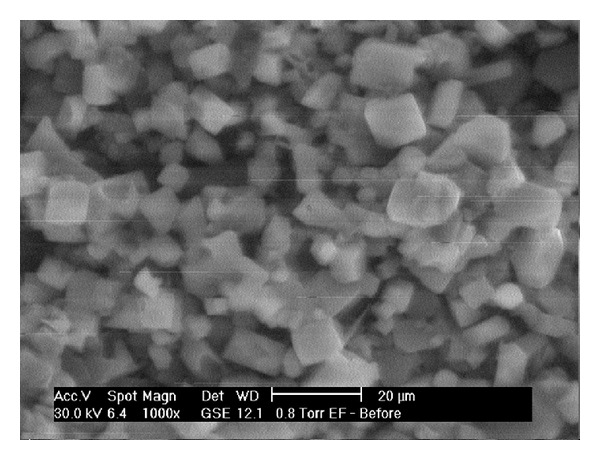
SEM photograph of MHF (1000x).

**Figure 8:**
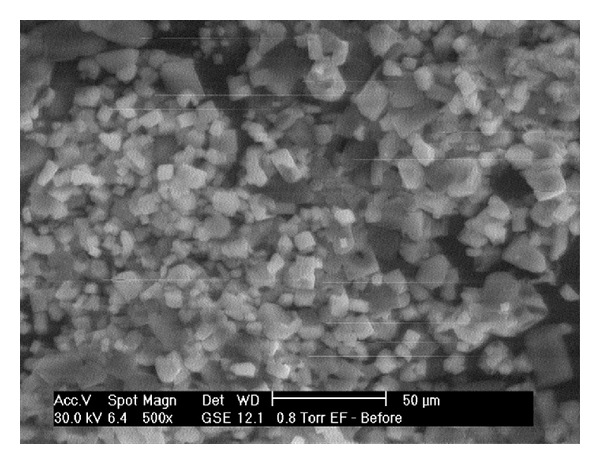
SEM photograph of MHF (500x).

The colour of all the MHFs observed by the naked eyes was white. The SEM images of MHFs are given in Figures 7 and 8 at 1000x and 500x, respectively. The crystals of the ECN were clearly observed on the surface of MHF.

### 3.3. Drug Content Uniformity Studies

The results of drug content uniformity are given in the Table 4. The drug content of the MHFs was found in the range 98.98 ± 0.91% to 100.45 ± 0.45%.

### 3.4. Physical Properties of MHFs

The results of microenvironment pH, thickness, weight, and folding endurance of the MHFs are given in the Table 4. The microenvironment pH of the MHFs was between 7.21 ± 0.14 and 7.41 ± 0.18. The microenvironment pH was unaffected by concentration of PVA and number of freeze/thaw cycles. The pH of all the MHFs was near to the pH range of the oral cavity. So, there is minimum possibility of the irritation to the oral mucosa after application. The MHF thickness was found in between 0.32 ± 0.07 mm (EF3) to 0.61 ± 0.05 mm (EF7). As the concentration of PVA increased the thickness of the MHFs increased. This is because PVA is the major part of the MHF and increase in its concentration leads to increase in thickness. The MHF weight was found in between 194 ± 1.41 mg (EF1) and 228 ± 2.05 mg (EF8). As the concentration of PVA increased the weight of the MHFs increased. This is because PVA is the major part of the MHF and increase in its concentration leads to increase in weight. The folding endurance was more than 300 number of folding for all the formulations. This indicates very high physical strength of the MHFs.

### 3.5. Moisture Absorption

The results of the moisture absorption studies are given in the Table 5. The percent moisture absorption increased as the concentration of PVA increased. This may be due to increase in the water absorbing PVA molecules by increasing the concentration of PVA. But there was no effect of number of freeze/thaw cycles on the percent moisture absorption. The difference in the moisture absorption from day 3 to day 7 is very less in all the formulations. This may be because of the saturation of the MHF with the moisture absorption within 3 days. This is done to evaluate the moisture absorption ability of the MHFs so that precautions can be taken to avoid it.

The highest percent moisture absorption for the MHFs for day 1, day 3, and day 7 was found to be 29.14 ± 1.06, 32.44 ± 1.85 and 32.65 ± 2.03 percent, respectively, for the formulation EF7 and lowest percent moisture absorption was 19.24 ± 1.36, 22.05 ± 1.14 and 22.12 ± 1.47 percent, respectively, for the formulation EF1.

### 3.6. In Vitro Mucoadhesive Studies

**Figure 9:**
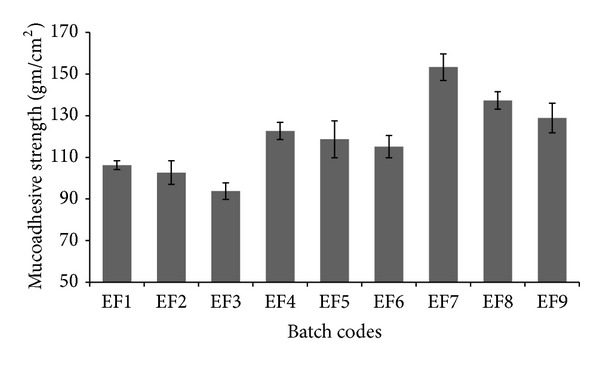
In vitro mucoadhesive strength (gm/cm^2^) of MHFs.

The mucoadhesive studies were expressed in gm/cm^2^ of the mucoadhesive strength (MS). The results of the in vitro mucoadhesive studies are given in the Table 4 and Figure 9.

As the concentration of PVA increased the MS increased and there was decrease in the MS as the number of freeze/thaw cycle increased within the same concentration of PVA. This observation agrees with that of the Peppas and Mongia [15] and Tsutsumi et al. [38]. The reason may be the same as explained by the above workers that the work of fracture decreased with increasing number of freezing/thawing cycles. This observation is attributed to loss of adhesive linear PVA chains as crystallization occurs due to the slow incorporation of all the linear PVA chains in the crystalline structure formed upon repeated cycles. Thus, a hydrogel produced after four cycles contains relatively mobile, noncrystalline chains which exhibit strong adhesive behavior either because of hydrogen bonding due to their hydroxyl groups because of significant chain interpenetration or because of both. Contrary to this, after six and eight cycles, very few linear PVA chains are available for this interaction with mucin.

The highest and lowest MS were found to be 153.33 ± 7.09 gm/cm^2^ (EF7) and 93.78 ± 2.12 gm/cm^2^ (EF3), respectively.

### 3.7. Water Uptake or Swelling Study

**Figure 10:**
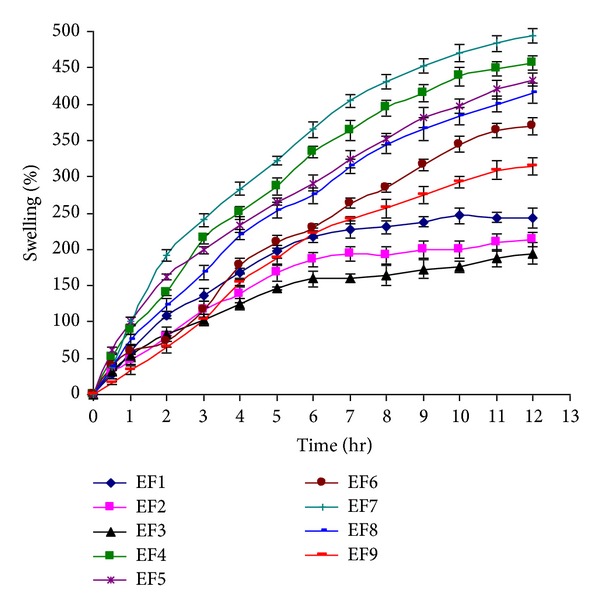
Percent swelling versus time curve.

The results of the water uptake or percent swelling are given in Figure 10. The highest swelling at twelfth hour was found to be 493.94 ± 10.25 percent (EF7) and lowest swelling at twelfth hour was 193.91 ± 14.78 percent (EF3). It was found that as the concentration of PVA increased the percent swelling increased significantly (*P* < 0.05). This is because the increase in the water absorbing PVA molecules by increase in concentration of PVA. It was observed that within the same concentration of PVA, as the number of freeze/thaw cycles increased, the percent swelling was decreased significantly (*P* < 0.05). The reason for this behavior may be the increase in the crosslinking or the degree of crystallinity, by increase in the freeze/thaw cycles [15, 17, 20, 39].

The percent swelling or the water uptake data were subjected to the Vergnaud model to determine the rate of water uptake and to study the polymer relaxation and the drug release mechanism. According to Ebube et al., 1997 [33], a value of ≤0.5 for *n* indicates a diffusion controlled mechanism in which the rate of diffusion of the liquid is much less as compared with the rate of relaxation of the polymer segment. A value of one for *n* (*n* = 1) suggests that the stress relaxation process is very slow as compared with the rate of diffusion. This means that the liquid diffuses through the polymer matrix at a constant velocity showing an advancing front marking the limit of liquid penetration. A value of *n* between 0.45 and 1 indicates an anomalous or complex behavior in which the rate of diffusion of the liquid and relaxation of polymeric chains are of the same magnitude.

Table 6 shows that in all cases *n* lies in the range 0.56 < *n* < 0.95, which is indicative of an anomalous mechanism of water uptake in which solvent diffusion, as well as polymer relaxation, are of the same magnitude.

### 3.8. Determination of the In Vitro Residence Time

The results of the in vitro residence time are given in the Table 4. The mucoadhesive and in vitro residence studies are similar on the generalized view but the major difference is that in mucoadhesive studies the force in mg/cm^2^ required to detach the MHF from the mucosa surface was considered whereas the in vitro residence time is the time required to detach the MHF from the mucosa surface with continuous motion in the pH 6.8 phosphate buffer. So, in later case, the swelling, water penetration into the polymer matrix, and the motion are the cause of the detachment and simulates the environment of the oral cavity.

The in vitro residence time increased as the concentration of the PVA increased. It was observed that the residence time was decreased by increase in the number of freeze/thaw cycles within the same concentration of PVA. The reason behind this behaviour may be the same as for the mucoadhesive studies but one more factor to be considered for this case is the water uptake. The increase in number of freeze/thaw cycles causes increase in crosslinking and the degree of crystallinity [15, 17, 20]. Hydrogels produced after four cycles contain relatively mobile, noncrystalline chains which exhibit strong adhesive behavior either because of hydrogen bonding due to their hydroxyl groups or because of significant chain interpenetration or because of both. Contrary to this, after six and eight cycles, very few linear PVA chains are available for this interaction with mucin. And percent swelling also decreases with increasing the number of freeze/thaw cycles due to the reason discussed under swelling studies. Because of all of the above reasons the in vitro residence time decreased by increasing number of freeze/thaw cycles. The highest and lowest in vitro residence time were found to be 734 ± 47 minutes (EF7) and 298 ± 21 minutes (EF3), respectively.

Most of the MHFs, especially containing 15% and 20% PVA, shown the higher than the required in vitro residence time, that is, >12 hours or >720 minutes.

### 3.9. In Vitro Drug Release Study

#### 3.9.1. Effect of Concentration of PVA on the ECN Release

**Figure 11:**
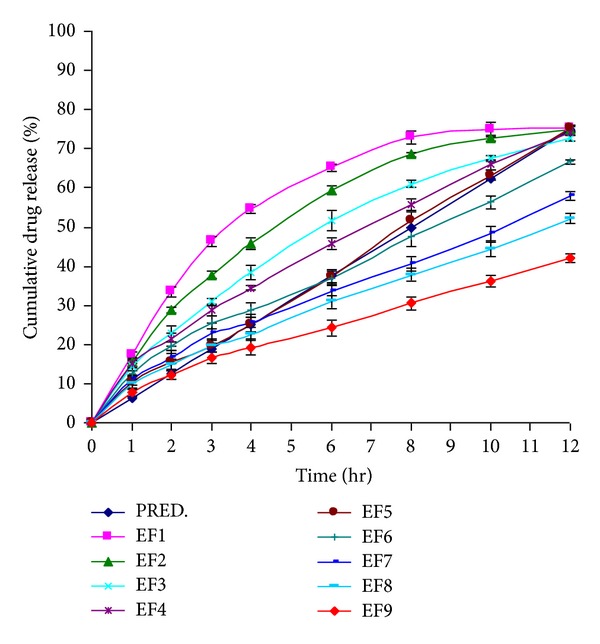
In vitro cumulative percent of drug release versus time curve (PRED: percentage predicted (theoretical) drug release profile).

The cumulative percent ECN release at different time intervals is given in Figure 11. Because of its poor aqueous solubility, the ECN was incorporated as solid dispersion (using ethanol and PEG400 3 : 5) into the MHFs, to increase its dissolution. But it was found that the maximum dissolution was raised to 66.66 *μ*g/mL after incorporating ECN as solid dispersion. The volume of dissolution media (pH 6.8 phosphate buffer with 1% SLS) used was 900 mL. It was observed that the maximum ECN release from the MHFs after an excessively long time of dissolution was found to be ~75% irrespective of the formulation parameters. So, 75% ECN was taken as the predicted value for the maximum release.

As the concentration of PVA increased, the ECN release decreased significantly (*P* < 0.05). The reason behind this type of release behavior may be the same as described by various workers. The higher the concentration of PVA, the higher the degree of crystallinity [22] and the higher the PVA network system or the denser the crosslinking [18, 19]. The above reasons cause the firm entrapment of the ECN into the PVA matrix network at higher PVA concentrations which leads to the decrease in the release.

The release profiles were studied for zero order kinetics, Korsmeyer-Peppas equation, Higuchi square root of time model and first order kinetics to determine the mechanism of drug release. The kinetic constants are given in the Table 7.

Best fit was observed with Korsmeyer-Peppas equation with *R* ^2^ > 0.99 excluding EF1, EF2. It clearly indicates that, at lower concentration, that is, 10% of PVA, release profile showed poor fit with Korsmeyer-Peppas equation model. According to the Korsmeyer-Peppas equation, *n* = 0.5 indicates Fickian release (diffusionally controlled release) and *n* = 1 indicates a purely relaxation controlled delivery which is referred to as Case II transport. Intermediate values (0.5 < *n* < 1) indicate an anomalous behavior (non-Fickian kinetics corresponding to coupled diffusion/polymer relaxation) [40]. Occasionally, values of *n* > 1 have been observed, which has been regarded as Super Case II kinetics [41, 42].

The values 0.58 < *n* < 0.83 of Korsmeyer-Peppas equation indicate the anomalous behavior, that is, non-Fickian kinetics corresponding to coupled diffusion/polymer relaxation.

The release behavior showed poor fit with Higuchi square root of time model (*R* ^2^ < 0.99 except EF3 and EF4), so the release mechanism is again concluded to be non-Fickian diffusion. The release behavior showed poor fit with first order kinetics (*R* ^2^ < 0.99 except EF3 and EF4) and zero order kinetics (*R* ^2^ < 0.99 except EF5).

#### 3.9.2. Effect of Number of Freeze/Thaw Cycles on the ECN Release

As the number of freeze/thaw cycles increased the ECN release decreased significantly (*P* < 0.05), within the same concentration of PVA. The results are similar as observed by various workers [15, 43]. The reason behind this type of release behavior maybe the densification of the crystalline structure [43], increasing the crosslinking network or high degree of crystallinity [21] by increasing the number of freezing/thawing cycles, thus leading to a reduction of ECN release.

The maximum release of ECN at twelfth hour was found to be 75.12 ± 1.02% for EF1.

The ECN release for EF5 to EF9 was less than 75% at twelfth hour but when the dissolution was done for more than twelve hours, it was observed that the maximum ECN release was ~75%.

At 10% PVA as the number of freezing/thawing cycles increased, the diffusion coefficient or the *n* of the Korsmeyer-Peppas equation increased (Table 7). But at 15 and 20% PVA there was no significant increase in the *n* value noticed. The number of freeze/thaw cycles or the degree of crystallinity has no effect on the mechanism of drug release.

### 3.10. Optimization of the MHFs

The optimization was done by 3^2^ full factorial design using response surface methodology (RSM). The independent variables used were concentration of PVA (% w/v of the initial gel) (*X*_1_) and number of freeze/thaw cycles (*X*_2_). The dependent variables used were time required for 50% drug release (*Y*_1_), percent of drug release at 8th hour (*Y*_2_), and “*k*” of zero order equation (*Y*_3_).

#### 3.10.1. Optimization of the Time Required for 50% ECN Release (*T*_50%_) (*Y*_1_)

The time required for 50% ECN release (*T*_50__%_) showed correlation coefficient, *R* ^2^ value, of 0.99, indicating a good fit. The *T*_50__%_ value for the nine batches (EF1 to EF9) showed a wide variation (3.44 hours to 14.21 hours). The data clearly indicate that the *T*_50__%_ values are strongly dependent on the selected variables.

In order to determine the levels of factors which yield optimum dissolution responses, polynomial regression equation was generated between the dependent and independent variables using Microsoft Excel. The responses of the *T*_50__%_ are given in (6). The polynomial equation can be used to draw conclusions after considering the magnitude of coefficient and the mathematical sign it carries, that is, positive or negative:

(6) Y=3.486−0.186∗X1−0.683∗X2+0.0384∗X1X2 +0.0232∗X12+0.0635∗X22.

**Figure 12:**
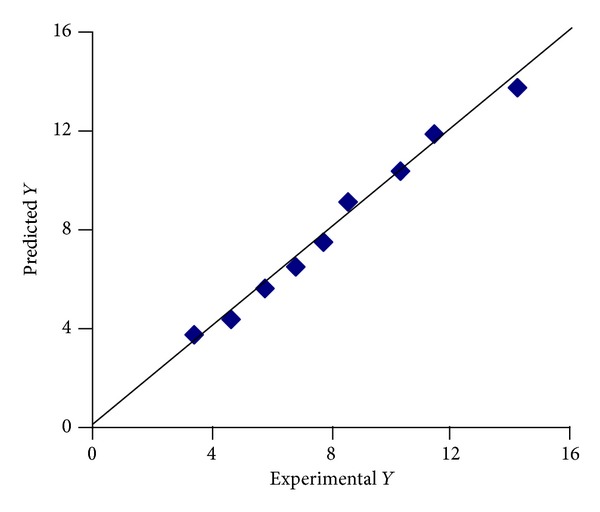
Percentage similarity of experimental and predicted values of optimization of the *T*_50%_.

A linear plot (Figure 12) drawn between the predicted and observed responses demonstrates high values of *R* ^2^ (*R* ^2^ > 0.9), indicating excellent fit. Thus low residuals as well as the significant value of *R* ^2^ designate a high prognostic ability of response surface methodology.

**Figure 13:**
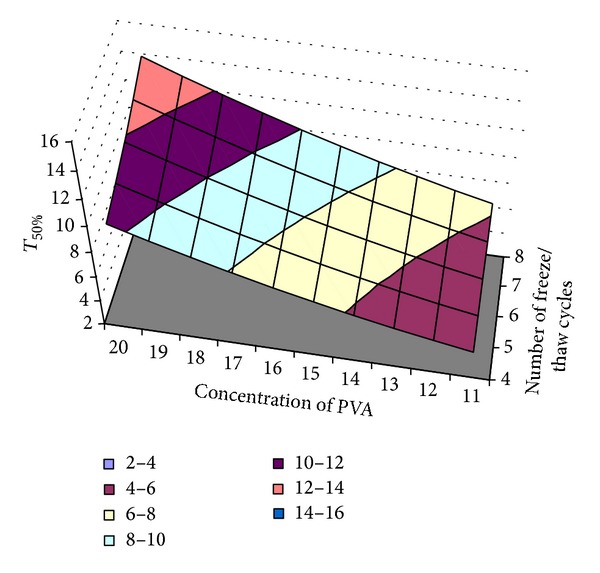
Surface plot of the optimization of *T*_50%_.

Figure 13 shows the surface plot of *T*_50__%_ (*Y*_1_) versus concentration of PVA (*X*_1_) and number of freeze/thaw cycles (*X*_2_). The plots were drawn using Microsoft Excel software. The data demonstrate that both the factors (*X*_1_ and *X*_2_) affect the drug release (*T*_50__%_). It may also be concluded from the graph that the high level of *X*_1_ (concentration of PVA) and high level of *X*_2_ (number of freeze/thaw cycles) favor the sustained release of the ECN from the MHFs, that is, increase in the *T*_50__%_. The low value of *X*_1_ *X*_2_ coefficient of (6) also suggests that the interaction between *X*_1_ and *X*_2_ is not significant.

#### 3.10.2. Optimization of Cumulative Amount of ECN Release at 8th Hour (Rel_8 hr_) (*Y*_2_)

The percent ECN release at 8th hour (Rel_8 hr_) showed correlation coefficient, *R* ^2^ value, of 0.997, indicating a good fit. The Rel_8 hr_ value for the nine batches (EF1 to EF9) showed a wide variation (30.54% to 72.95%). The data clearly indicate that the Rel_8 hr_ values are strongly dependent on the selected variables.

The responses of the Rel_8 hr_ are given in

(7) Y=109.354−3.712∗X1+0.639X2+0.045∗X1X2 +0.0109∗X12−0.320∗X22.

**Figure 14:**
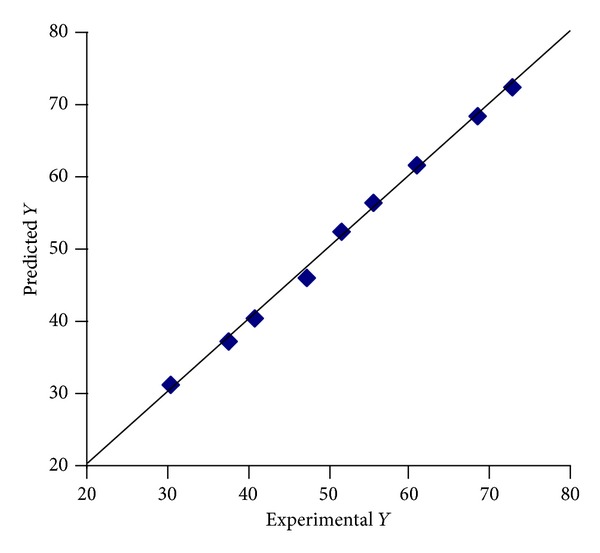
Percentage similarity of experimental and predicted values of optimization of the Rel_8 hr_.

A linear plot (Figure 14) drawn between the predicted and observed responses demonstrates high values of *R* ^2^ (*R* ^2^ > 0.9), indicating excellent fit. Thus low residuals as well as the significant value of *R* ^2^ designate a high prognostic ability of response surface methodology.

**Figure 15:**
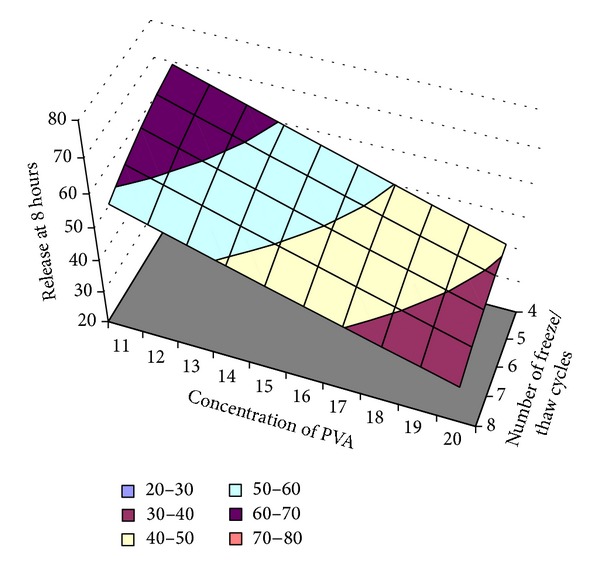
Surface plot of the optimization of the Rel_8 hr_.

Figure 15 shows the surface plot of Rel_8 hr_ (*Y*_2_) versus concentration of PVA (*X*_1_) and number of freeze/thaw cycles (*X*_2_). The data demonstrate that both the factors (*X*_1_ and *X*_2_) affect the drug release (Rel_8 hr_). It may also be concluded from the graph that the high level of *X*_1_ (concentration of PVA) and high level of *X*_2_ (number of freeze/thaw cycles) favor the sustained release of the ECN from the MHFs, that is, decrease in the Rel_8 hr_. The low value of *X*_1_ *X*_2_ coefficient of (7) also suggests that the interaction between *X*_1_ and *X*_2_ is not significant.

#### 3.10.3. Optimization of “*k*” of Zero Order Equation (*Y*_3_)

The “*k*” of zero order equation showed correlation coefficient, *R* ^2^ value, of 0.997, indicating a good fit. The “*k*” of zero order equation value for the nine batches (EF1 to EF9) showed a wide variation (3.79 to 8.20). The data clearly indicate that the “*k*” of zero order equation values are strongly dependent on the selected variables.

The responses of the “*k*” of zero order equation are given in (8). The *β*_1_ coefficient is positive (0.0192), indicating that “*k*” of zero order equation increases by increasing the concentration of PVA, and the value of *β*_2_ is also positive (0.0973), indicating that the “*k*” of zero order equation increases by increasing the number of freeze/thaw cycles:

(8) Y=9.194+0.0192∗X1+0.0973∗X2−0.00566∗X1X2 −0.0100∗X12−0.0245∗X22.

**Figure 16:**
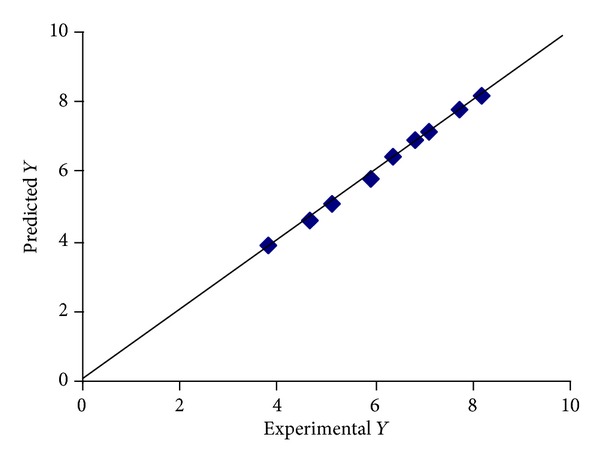
Percentage similarity of experimental and predicted values of optimization of the “*k*” of zero order equation.

A linear plot (Figure 16) drawn between the predicted and observed responses demonstrate high values of *R* ^2^ (*R* ^2^ > 0.9), indicating excellent fit.

**Figure 17:**
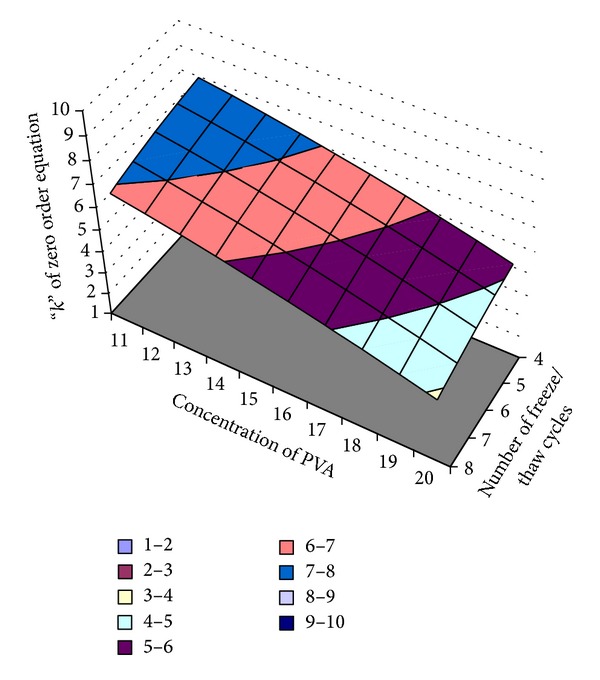
Surface plot of the optimization of the “*k*” of zero order equation.

Figure 17 shows the surface plot of “*k*” of zero order equation (*Y*_3_) versus concentration of PVA (*X*_1_) and number of freeze/thaw cycles (*X*_2_).

## 4. Evaluation of Optimized MHF

The MHF optimized by the 3^2^ full factorial design with the required values of independent variables was prepared. The optimized MHF contains 15% PVA with 7 freeze/thaw cycles.

The optimized MHF was prepared and studied for various physical and physicochemical properties. The mucoadhesive strength and in vitro residence time of the optimized formulation were 126.23 ± 8.56 gm/cm^2^ and 736 ± 28 minutes, respectively. The in vitro residence time is higher than the required, that is, 12 hours. The optimized MHF was found to possess excellent mechanical strength and the firmness, that is, folding endurance >300 number of folding.

The water uptake data exhibited an excellent fit in the model with the value of exponent, *n*, 0.64 with *R* ^2^ = 0.998. The *n* value indicates an anomalous mechanism of water uptake in which solvent diffusion, as well as polymer relaxation, is of the same magnitude.

The optimized formulation was fitted well in the zero order equation (*R* ^2^ > 0.99).

Table 8 shows the antifungal activity of the aliquot sample against* C. albicans*. The drug released from the optimized MHF was able to inhibit the growth of* C. albicans* for 12 hours. The maximum diameter of zone of inhibition by the aliquots of optimized formulation was found to be 16.57 ± 0.59 mm at twelfth hour. From the standard calibration curve of ECN agar diffusion assay, the growth inhibition zone of 12 hr dissolution sample of optimized batch corresponds to 75.65 ± 0.67% of ECN, which correlated well with dissolution studies data.

## 5. Conclusion

It was concluded that the prepared MHFs possess good mucoadhesion and reasonable in vitro residence time which is important for prolonging the contact time of the drug with the buccal mucosa, thus improving the overall therapy of oral candidiasis. Mucoadhesive hydrogel film with 15% polyvinyl alcohol and 7 freeze/thaw cycles was optimized by comparing the required drug release rate using polynomial equation generated by running the nine sets of experiments. The optimized batch exhibited the sustained release of drug up to 12 hours with the good fit with zero order kinetics. The in vitro antifungal studies revealed that the drug released from the optimized MHF could inhibit the growth of* Candida albicans* for prolonged period up to 12 hours suggesting better patient compliance and higher therapeutic efficacy.

## Figures and Tables

**Table 1:** Batch codes and coded values of factor *X*_1_ and factor *X*_2_.

Batch code	Factor *X*_1_	Factor *X*_2_
EF1	−1	−1
EF2	−1	0
EF3	−1	+1
EF4	0	−1
EF5	0	0
EF6	0	+1
EF7	+1	−1
EF8	+1	0
EF9	+1	+1

**Table 2:** Coded values and actual values of factor *X*_1_ and factor *X*_2_.

Coded Values	Actual Values	
	Factor *X*_1_	Factor *X*_2_
−1	10	4
0	15	6
+1	20	8

*X*_1_: concentration of PVA (% w/v of film forming gel).

*X*_2_: number of freeze/thaw cycles.

*X*_1_ and *X*_2_ are independent variables.

The dependent variables are *Y*_1_, *Y*_2_, and *Y*_3_, where *Y*_1_ is time required for 50% drug release;

*Y*_2_ is percent of drug release at 8th hour;

*Y*_3_ is “*k*” of zero order equation.

**Table 3:** Formulae of MHFs.

Batch number	Econazole nitrate (mg per MHF)	PVA (% w/v of film forming gel)	Number of freeze/thaw cycles	PEG400 (% v/v of film forming gel)	Ethanol (% v/v of film forming gel)
EF1	80	10	4	5	3
EF2	80	10	6	5	3
EF3	80	10	8	5	3
EF4	80	15	4	5	3
EF5	80	15	6	5	3
EF6	80	15	8	5	3
EF7	80	20	4	5	3
EF8	80	20	6	5	3
EF9	80	20	8	5	3

**Table 4:** Physical properties of MHFs.

Batch code	Drug content* (%)	Microenvironment pH*	MHF thickness* (mm)	MHF weight* (mg)	Folding endurance*	Mucoadhesive strength** (gm/cm^2^)	In vitro residence time** (minute)
EF1	99.21 ± 0.94	7.32 ± 0.12	0.32 ± 0.08	194 ± 1.41	>300	106.22 ± 3.98	334 ± 34
EF2	100.01 ± 0.45	7.30 ± 0.22	0.33 ± 0.05	196 ± 2.12	>300	102.67 ± 5.67	321 ± 19
EF3	99.34 ± 0.65	7.21 ± 0.14	0.32 ± 0.07	195 ± 1.87	>300	93.78 ± 2.12	298 ± 21
EF4	99.78 ± 0.87	7.29 ± 0.19	0.48 ± 0.08	211 ± 0.80	>300	122.67 ± 5.34	469 ± 38
EF5	98.98 ± 0.91	7.36 ± 0.20	0.47 ± 0.11	213 ± 0.68	>300	118.67 ± 8.90	421 ± 41
EF6	100.45 ± 0.45	7.39 ± 0.12	0.47 ± 0.12	213 ± 1.09	>300	115.11 ± 4.12	409 ± 25
EF7	99.16 ± 0.73	7.40 ± 0.25	0.61 ± 0.05	226 ± 1.06	>300	153.33 ± 7.09	734 ± 47
EF8	99.56 ± 0.56	7.41 ± 0.18	0.60 ± 0.11	228 ± 2.05	>300	137.33 ± 4.23	718 ± 24
EF9	98.99 ± 0.90	7.40 ± 0.26	0.61 ± 0.03	227 ± 1.15	>300	128.89 ± 6.39	689 ± 30

*n = 5.

**n = 3.

**Table 5:** Percent moisture absorption in percentage.

Batch number	Percent moisture absorption*		
	Day 1	Day 3	Day 7
EF1	19.24 ± 1.36	22.05 ± 1.14	22.12 ± 1.47
EF2	20.14 ± 1.05	21.65 ± 1.35	21.89 ± 2.45
EF3	20.32 ± 1.87	22.17 ± 2.03	22.54 ± 1.44
EF4	23.58 ± 2.04	25.45 ± 0.86	25.96 ± 0.75
EF5	24.12 ± 0.89	26.14 ± 2.35	26.45 ± 1.24
EF6	24.87 ± 1.35	26.54 ± 1.42	26.58 ± 0.69
EF7	29.14 ± 1.06	32.44 ± 1.85	32.65 ± 2.03
EF8	28.65 ± 2.01	31.65 ± 2.25	31.67 ± 1.32
EF9	28.87 ± 2.35	32.24 ± 1.05	32.33 ± 1.85

*n = 3.

**Table 6:** Values of *n* and *k* of the Vergnaud model swelling kinetic equation.

Batch code	*n*	*k*	*R* ^2^
EF1	0.66	58.18	0.949
EF2	0.63	51.71	0.969
EF3	0.56	53.05	0.977
EF4	0.70	89.60	0.992
EF5	0.61	99.49	0.997
EF6	0.76	58.05	0.978
EF7	0.76	89.52	0.957
EF8	0.74	72.18	0.992
EF9	0.95	34.88	0.990

**Table 7:** Kinetic constants, time required to 50% ECN release (*T*_50%_), and ECN release at 8th hour (Rel_8 hr_).

Kinetic profile of MHFs	Higuchi eq.	Korsmeyer-Peppas eq.			First order eq.		Zero order eq.		*T*_50%_ (hr)	Rel_8 hr_ (CPR)
	*R* ^2^	*n*	*k*	*R* ^2^	*k*	*R* ^2^	*k*	*R* ^2^		
Desirability	0.977	1.00	6.25	1.000	−0.04	0.949	6.25	1.000	8.00	50.00
EF1	0.925	0.58	21.17	0.929	−0.06	0.876	8.20	0.623	3.44	72.95
EF2	0.976	0.63	17.62	0.973	−0.06	0.960	7.75	0.782	4.62	68.52
EF3	0.995	0.67	14.60	0.995	−0.05	0.993	7.09	0.884	5.75	60.95
EF4	0.993	0.66	14.17	0.997	−0.05	0.993	6.82	0.930	6.86	55.65
EF5	0.963	0.83	8.93	0.990	−0.04	0.954	6.34	0.995	7.76	51.76
EF6	0.980	0.65	12.27	0.994	−0.04	0.984	5.90	0.940	8.57	47.48
EF7	0.984	0.66	10.70	0.996	−0.03	0.983	5.13	0.933	10.36	40.74
EF8	0.987	0.67	9.45	0.996	−0.03	0.988	4.66	0.943	11.45	37.72
EF9	0.989	0.67	7.64	0.998	−0.02	0.979	3.79	0.936	14.21	30.54

**Table 8:** Diameter of zone of inhibition of optimized MHF.

Time (hr)	0	1	2	3	4	6	8	10	12
Diameter of zone of inhibition* (mm)	0	2.11 ± 0.56	3.24 ± 0.56	4.21 ± 0.24	5.44 ± 0.09	8.24 ± 0.79	11.39 ± 0.70	14.16 ± 0.82	16.57 ± 0.59
Corresponding percent drug release*	0	9.63 ± 0.89	14.79 ± 0.81	19.22 ± 0.33	24.84 ± 0.19	37.62 ± 1.08	52.00 ± 0.99	64.65 ± 1.14	75.65 ± 0.67

*n = 3.
